# Analysis of time-dependent effects of ambient temperatures on health by vulnerable groups in Korea in 1999–2018

**DOI:** 10.1038/s41598-023-28018-z

**Published:** 2023-01-17

**Authors:** Jongchul Park, Yeora Chae

**Affiliations:** 1 Kongju National University, 56 Gongjudaehak-ro, Gongju, 32588 Korea; 2 Korea Environment Institute, 370 Sicheong-daero, Sejong, 30147 Korea

**Keywords:** Health policy, Climate-change adaptation, Climate-change impacts, Climate-change policy, Sustainability

## Abstract

This study compared the relative risks of heat days on mortalities by vulnerable groups (elderly, single-person households, less-educated) in the past decade (1999–2008) and the recent decade (2009–2018) in four cities, Seoul, Incheon, Daegu, and Gwangju, in Korea. It has been known that the health impacts of heatwaves have gradually decreased over time due to socio-economic development, climate adaptation, and acclimatization. Contrary to general perception, we found that the recent relative risk of mortality caused by heat days has increased among vulnerable groups. It may associate with recent increasing trends of severe heat days due to climate change. The increasing relative risk was more significant in single-person households and less-educated groups than in the elderly. It implies that the impacts of climate change-induced severe heat days have been and will be concentrated on vulnerable groups. It suggests that social polarization and social isolation should be addressed to reduce heatwave impacts. Furthermore, this study shows the necessity of customized heatwave policies, which consider the characteristics of vulnerable groups.

## Introduction

The association between heatwave and the risks of disease and death has been proven in numerous studies^1–6^. The intensity and duration of heatwaves in the future are predicted to increase due to climate change^7^. The heatwave-related socioeconomic damages such as cases of illness, mortality, and health cost (socioeconomic cost) are also predicted to increase^8,9^. Understanding and adapting to climate change is a difficult predicament ahead of humanity. Many studies have investigated health regarding heatwaves and contributed to developing measures to effectively reduce its effects on human health. Previous studies explored concerns, including identifying heatwave-vulnerable groups^10–14^, the difference in threshold temperature concerning vulnerable groups and health indicators^6^, and the spatial difference in risks^3,15^.

Several studies have focused on the time-dependent variation in the effects of a heatwave. The relationship between heatwave and human health has continuously varied with time. This is a critical aspect of future risks assessment of climate change, the selection of vulnerable groups and regions, and the analysis of the impact prediction effects^11,16^. According to Gasparrini et al., time-dependent variation was observed for the risk of heatwave-related mortality in 272 regions across seven countries^17^. During 1985–2012, the risk of heatwave-related mortality decreased in the United States, Japan, and Spain. Chung et al. also report a fall in the risk of heatwaves in Japan compared to the past^18^. Lee et al. addressed that the risk of heatwaves decreased in several cities in Japan, Taiwan, and South Korea. In South Korea, 2018 heatwaves recorded the historical worst since 1994 events^19^. Nevertheless, excess mortality in 2018 due to heatwave was significantly reduced compared to 1994^20^.

Many factors could induce the time-dependent variation in the risk of heatwaves. The influencing factors reported by previous studies included improved healthcare resources such as the number of hospital beds^21^, the increase in evasive actions in consideration of the risk of heatwaves^22,23^, the use of such adaptive strategies as air conditioners^24–26^, and the policy effects on the heatwave warning system^27,28^. Many policies have been implemented to reduce damage from heatwaves in Korea since 2008. For example, services such as Heatwave warning system and emergency text message, service to make a phone call to the elderly, heat-related illness surveillance system, 119 heatwave ambulance, and the hot environment management guidelines have been implemented after 2008. The Korea Meteorological Administration has been making impact forecasts since 2019. Notably, the current data suggest that the provision of relevant risk information can effectively elicit evasive actions and enhance risk perception^23,29–31^.

The time-dependent variation in the risk of heatwaves has not yet been examined across vulnerable groups. Previous studies reported that the relatively vulnerable socioeconomic groups from heatwaves include the elderly, outdoor workers, individuals with chronic disease, low-income, and single-person household individuals^10–14^. The lack of change or increase in the time-dependent variation for the risk of heatwaves in vulnerable groups would imply that they are still facing difficulties with the perception of the risk of heatwaves and the performance of evasive actions. The relevant data can help improve heatwave-related policies. However, it is difficult to find a study on the temporal change of heatwave risk for vulnerable groups.

This study compared the differences in the risk of mortality caused by heat days between the past decade (1999–2008) and the recent decade (2009–2018) in four cities in South Korea. Heatwave is defined differently depending on the country and the region. The Korea Meteorological Administration issues a heatwave warning when the maximum temperature is expected to exceed 33 °C for two consecutive days. However, it is known that the impacts of high temperatures appear before that^6^. Therefore, in this study, the ranges of high temperatures were divided into Mild, Moderate, and Severe heat days from the threshold temperature which is the number of deaths began to increase. And the health impacts of each heat event were analyzed. The time-dependent variation in the risk of mortality caused by heat days in each vulnerable group (elderly, single-person households, and less-educated) was also analyzed.

## Study period and sites

This study analyzed the heterogeneous relative risk of heat-related mortality by time, vulnerable groups, and regions. We used two time periods, the past decade, 1999 to 2008, and the recent decade, 2009 to 2018. In South Korea, many heatwave-related policies have been implemented since 2008. For example, the heatwave warning system service and the emergency disaster text message service were initiated in 2008. Moreover, the automated-phone warning service for the elderly upon heatwave began in 2009. Thus, this study compared the risks of heatwave before and after 2008.

The study sites were the following four cities: Seoul, Incheon, Daegu, and Gwangju. Among these metropolitan cities in Korea, these are cities with high population (Seoul, Incheon) or high frequency of heatwaves (Gwangju, Daegu). Busan, Korea's second city, was excluded from the study because the relationship between ambient temperature and excess mortality was unclear in the previous study^20^. Table 1 shows the socioeconomic and climatic characteristics of the study sites. Especially, Seoul is the most densely populated city in South Korea and a megacity. The city with the most recent highest elderly percentage was Daegu (11.6%), and with the most prominently increased percentage than before was Seoul (increased from 6.5% to 11.3%). The percentage of low-income individuals was high in Gwangju and Daegu compared to Seoul and Incheon, and the percentage of single-person households was also high in Gwangju and Daegu.

**Table 1:** The population and climate of summer (JJA) in study cities.

Group	Period	Seoul	Incheon	Gwangju	Daegu
Population(million)	1999–2018	9.77	2.96	1.46	2.46
Elderly (%)	1999–2008	6.5	5.9	6.5	7.0
	2009–2018	11.3	9.4	10.4	11.6
Low-income (%)	2009–2018	2.4	2.9	4.9	4.3
Single-person (%)	2010–2018	10.5	8.1	10.4	9.3
Average of Tmax (°C)	1999–2008	28.5	27.8	29.2	29.7
	2009–2018	29.5	27.6	30.4	30.9
Average of Rh (%)	1999–2018	72.1	77.9	74.8	69.7
	2009–2018	69.2	82.7	76.6	67.8

*Tmax: maximum temperature; Rh: relative humidity.

**Source of data: Statistics Korea^32^.

Gwangju and Daegu have a higher maximum temperature (Tmax) in summer (June to August; JJA) than Seoul and Incheon. The average Tmax of Daegu is 30.9 °C, the highest among the study cities. Incheon showed lower Tmax than other metropolitan cities. Relative humidity (Rh) decreased in Seoul and Daegu, and increased from 77.9% to 82.7% in Incheon (Table 1).

**Figure 1:**
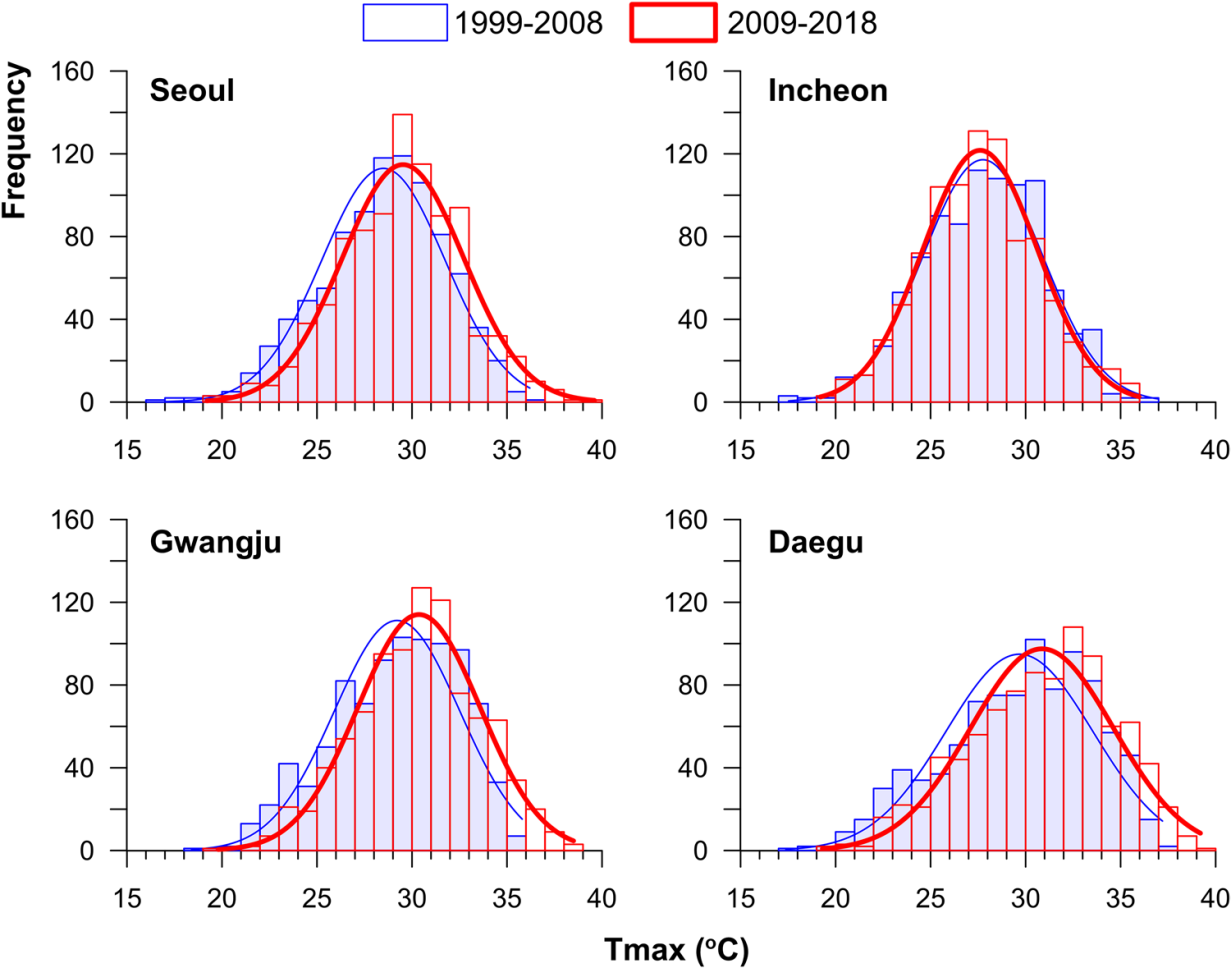
Comparison of the distribution of the Tmax during the summer (JJA) between the past (1999–2008) and the recent (2009–2018).

Except for Incheon, the three cities showed an increase in mean Tmax by approximately 1 °C between these two decades. In addition, the Tmax kernel density significantly shifted to the right. Incheon showed no significant change in the Tmax kernel density (Fig. 1).

## Materials and methods

### Data source and study population

The mortality data were obtained from the Microdata Integrated Service of Statistics Korea. The data contained information regarding the date and cause of death, city-level location of death, age at the time of death, occupation, and marital status. Total mortality was aggregated excluding external causes of death (International Classification of Diseases 10th Revision (ICD-10) codes A-R).

### Socioeconomic classification of the deceased

This study defined vulnerable groups as elderly, single-person household, and less-educated groups. These groups were considered as vulnerable groups because it is well known that they are relatively strongly affected by heat waves in previous studies^10,14^. The elderly were defined as individuals aged 65 years or above. The single-person household was defined as individuals aged 20 years or above at the time of death in the following status: unmarried, unknown marital status, divorced, or bereaved. The less-educated group was defined as the level of education at or below middle school graduation. The percentage of the elderly, single-person households, and less-educated group were 69.7%, 49.4%, and 65.8% of total mortality during the summer, respectively (The detailed number of deaths according to socio-economic conditions can be found as Supplementary Table S1).

### Ethical approval

This study protocol was reviewed and approved by the Institutional Review Board (IRB) of the Ministry of Health and Welfare of South Korea (Confirmation number P01-202107-22-007). As this was an observational study without intervention and de-identified statistical data was used, the requirement for informed consent was waived by the same IRB committee. All methods were carried out in accordance with Korean government’s guideline for health and medical data utilization.

### Weather data

The maximum and minimum temperature (Tmax, Tmin) and relative humidity (Rh) were obtained from the automated synoptic observing system (ASOS) of each metropolitan city, provided by the Korea Meteorological Administration^33^. In this study, heat days were defined as three types: Mild, Moderate, and Severe heat days. Mild heat days have Tmax in the 60th–90th percentile range. Moderate and Severe heat days have Tmax in the 90th–99th and 99th or more percentile ranges, respectively.

### Statistical analysis

A distributed lag non-linear model (DLNM) which is proposed by Gasparrini was used to analyze the relative risk of heat-related mortality^34^. We implemented the DLNM 2.4.7^35^ using dlnm package in R software version 4.1.1.

$$\ln \left( {{\text{E}}\left( {{\text{y}}_{{\text{t}}} } \right)} \right) = \alpha + \beta Tmax_{t,l} + NS\left( {rhavg_{t} } \right) + NS\left( {prcp_{t} } \right) + NS\left( {doy_{t} } \right) + NS\left( {sn_{t} } \right) + \gamma weekday_{t}$$

Here, ${\text{E}}\left( {{\text{y}}_{{\text{t}}} } \right)$ denotes the expected daily death counts on day t, which follows the Poisson distribution. $T{\text{max}}_{t,l}$ is the cross-basis of temperature and lag time l. the lag time was determined through AIC (Akaike information criterion) test (Table S2 and Figure S1). The $rhavg_{{\text{t}}}$ is the average Rh on day t, $prcp_{t}$ is the precipitation on day t, ${\text{doy}}_{{\text{t}}}$ is the day of the year on day t, and $sn_{t}$ is the sequential serial number of the date on day t. The serial number was used to adjust long-term trends in mortality. The weekday is a dummy variable for weekdays. NS denotes natural spline function.

The association between temperature and mortality was analyzed for each city, population group, and period (the past decade and the recent decade). Percent changes of relative risk (RR) per 1 °C were calculated for three temperature ranges (Mild, Moderate, and Severe heat days). The percent change of RR was calculated by the following formula.

$$Percent\;change = \frac{{\left( {{\text{RR}}_{n} - {\text{RR}}_{1} } \right) / \left( {n - 1} \right)}}{1}*100$$

Here, RR_1_ and RR_n_ are the first and the last of each temperature range, respectively.

## Results

**Figure 2:**
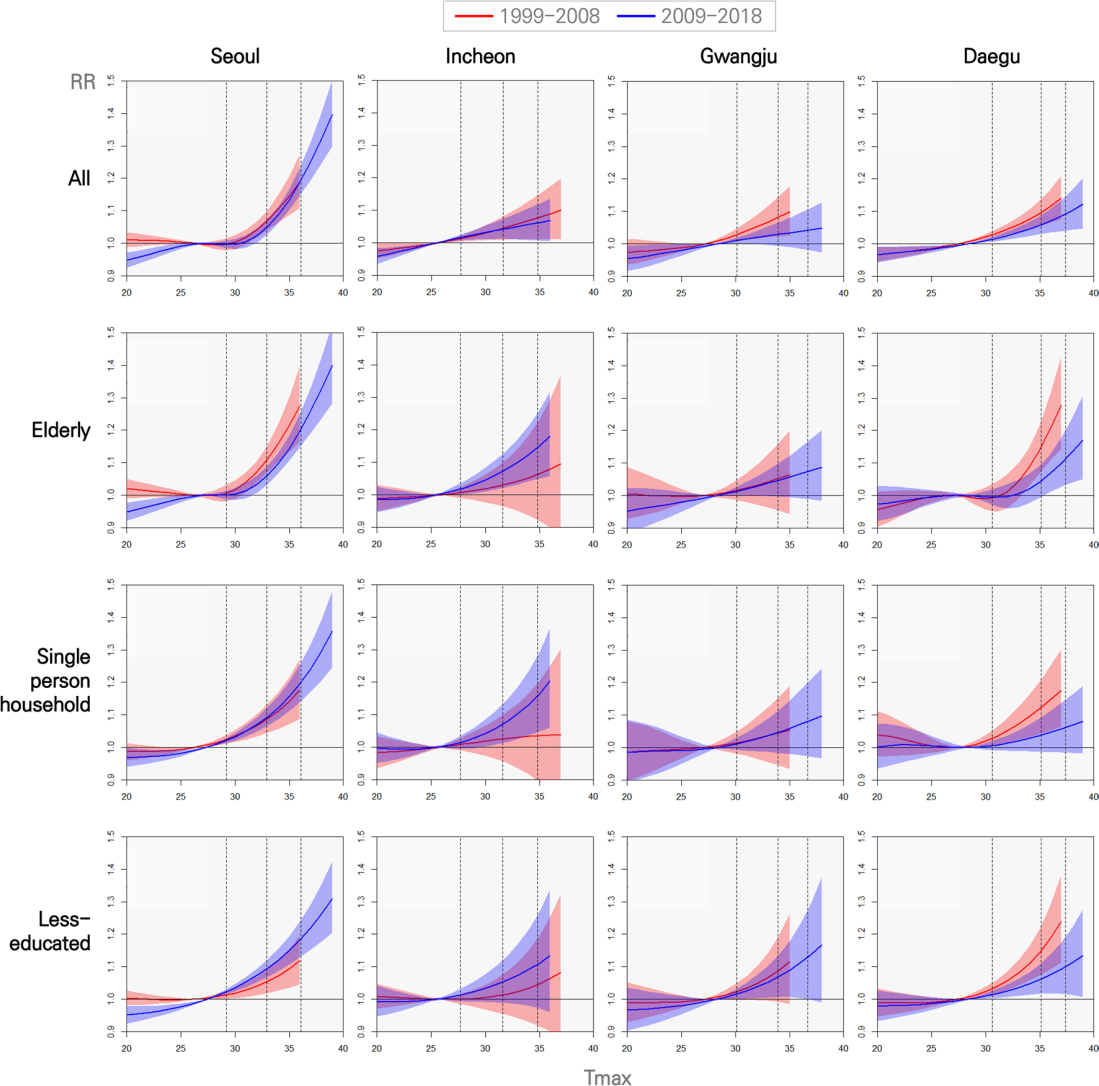
Comparison of associations between Tmax and mortality depending on vulnerable groups in the past and recent. The three vertical dotted lines indicate the 60, 90, and 99 percentile Tmax lines, respectively.

Figure 2 shows the temporal change in the association between temperature and mortality for the vulnerable groups. The health impacts of high temperature began to appear at the 60th percentile of the Tmax in summer. In all cities, the recent RR for all ages decreased compared to the past RR (All), but the recent RR of the vulnerable group increased in Seoul, Incheon, and Gwangju (elderly, single person household, and less-educated). In Daegu, the recent RR decreased compared to the past in all cases.

Recently, the RR of the vulnerable groups (elderly, single, and less-educated) increased in Seoul and Incheon. For single-person households and less-educated in Seoul, the RR increased in Mild to Severe heat days, but the RR of the elderly increased rapidly only at a severe temperature range. In Incheon, the recent RR has increased compared to the past among the elderly, single-person households, and less-educated groups. In Gwangju, the RR of single-person households increased slightly compared to the past. The RR of the less-educated group is increasing rapidly in the severe temperature range.

Table 2 quantifies the % rate of change in RR. In an All group, RR increased relatively rapidly in Moderate and Severe heat days, whereas RR decreased in all other cities compared to the past.

**Table 2:**
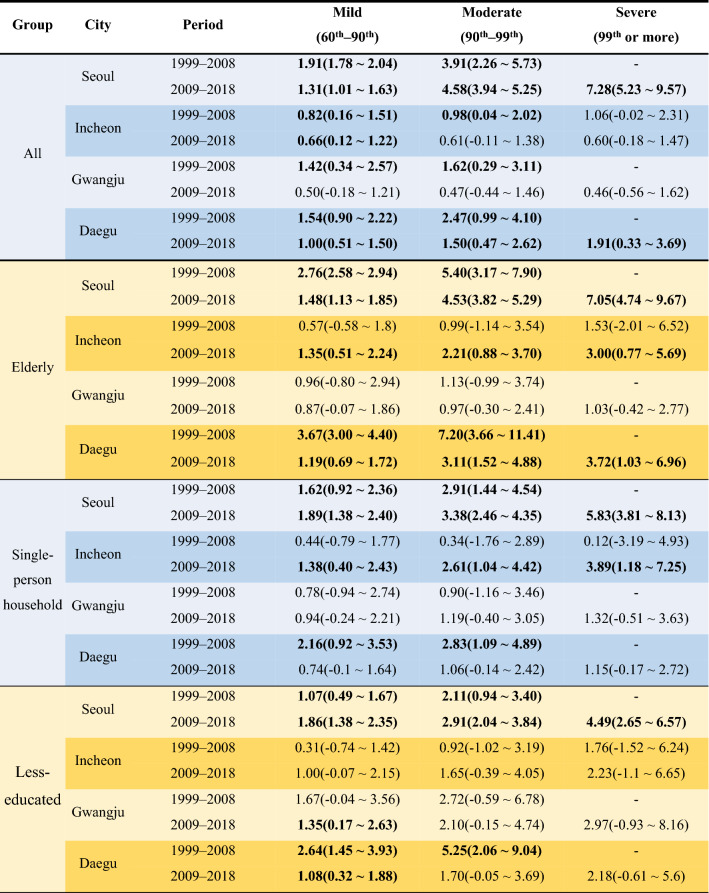
Percent change in relative risk for mortality at 95% confidence interval for three Tmax ranges.

‘–’ indicates the absence of the 99th percentile temperatures in the past (1999–2008).

Bold values mean that RR was increased statistically significantly.

In the elderly group of Seoul, RR increased by 7.05% (4.74 ~ 9.69) in the Severe heat days, and this value is 2.52% points higher than the RR in the Moderate heat days in the past. Incheon's RRs increased by 1.35% (0.51 ~ 2.24), 2.21% (0.88 ~ 3.70), and 3.00% (0.77 ~ 5.69) in Mild, Moderate, and Severe heat days, respectively, which were higher than in the past.

In 2009–2018, the RRs of single-person households in Seoul were increased by 1.89% (1.38 ~ 2.40), 3.38% (2.46 to 4.35), and 5.83% (3.81 ~ 8.13) in all three temperature ranges, respectively. In Incheon, the recent RRs increased by 1.38% (0.40 ~ 2.43), 2.61% (1.04 ~ 4.42), and 3.89% (1.18 ~ 7.25) in three temperature ranges, respectively. The increasing trends of relative risk are significantly higher than past. The increase rate of RR in Incheon was 2.27% point higher in the Moderate heat days and 3.77% point higher in the Severe heat days than in the past. The result of Gwangju shows that the RR of single-person households was slightly higher than in the past. The RRs were 0.94% (− 0.24 ~ 2.21), 1.19% (− 0.40 ~ 3.05), and 1.32% (− 0.51 ~ 3.63) in each range, respectively. The risk was in an increasing pattern with temperature, although it involves a high degree of uncertainty because the lower bound of 95% confidence interval was negative.

The less-educated group clearly showed an increase in RR recently compared to the past in Seoul. In the recent period, the RR increased by 2.91% (2.04 ~ 3.84) per 1 °C in the Moderate heat days, whereas in the past period, the RR increased by 2.11% (0.94 ~ 3.40). In the recent Severe heat days, RR increased rapidly by 4.49% (2.65 ~ 6.57). Recently in Incheon, although it was not statistically significant in the 95% significance interval, there was a pattern of increasing RR compared to the past. For example, in the Severe heat days, RR increased by 2.23% (− 1.1 ~ 6.65). In Gwangju, the recent RR increased by 2.97% (-0.93 ~ 8.16) in the same temperature range.

## Discussion

Under the climate change in the future, there is a possibility that heat events that we have not experienced so far will appear^36^. It is well known that negative outcomes increase dramatically when unusual heat events occur, for examples, the 2003 European heatwave^37^, the 1994 and 2018 heatwaves in Korea^20^, and the 1995 Chicago heatwave^38^. We found that negative outcomes caused by heatwaves can be reduced by various policies and socioeconomic development, but the negative outcomes of vulnerable groups such as single-person households and low-educated groups have increased rapidly during the Severe heat days, contrary to our expectations. It means that if the heatwave response policy is focused on the elderly in terms of age and does not fully consider socioeconomic conditions, negative health effects under future climate change may be concentrated on these vulnerable groups.

In order to reduce negative outcomes from heatwaves in socio-economically vulnerable groups, effective warning dissemination and communication, social risk avoidance systems (e.g., guidelines on work intensity levels) is required. In addition, more effective measures that can induce heat protection behaviors in vulnerable groups are needed. Several previous studies^27,28^ showed that heatwave warning systems contributed to reducing human casualties. Heatwave warning system and emergency text message service have been implemented in Korea since 2008. This system is expected to contribute to reducing the scale of human casualties caused by the 2018 heat wave compared to the 1994 heatwave^20^. However, this study shows the possibility that the risk communication through the warning system is not sufficient to induce heat protection behavior in socially isolated and less-educated groups. Vulnerable groups have difficulty obtaining information about the risk of heatwaves^39^ and lack awareness of the risk of heatwaves^23^. Low-education and low-income groups are less likely to have personal heat protection measures^40^. Therefore, it is necessary to understand which behavioral patterns of these vulnerable groups are causing risk in extreme heat waves and to develop policies that can disconnect the risk-generation process.

By the way, the less-education group used in this study can be regarded as a proxy variable for low-income group. Table 3 shows the relative wage index by education level. When the income of high school graduates is regarded as 100, it can be seen that the income increases as the level of education increases. Vulnerability in the less-educated group may be due to low income.

**Table 3:** Relative wage index by education in South Korea (1998–2018).

Less than middle school	High school	College	University
72.1%	100.0%	113.0%	152.9%

OECD ^41^.

The relative wage index is converted by taking the average wage of high school graduates as 100 (adult population aged 25–64).

The Tmax in Incheon in the summer was lower than in the past, while the Tmax has risen in the other three cities. Nevertheless, the relative risk of vulnerable groups has risen recently in Incheon. As a result of comparing the number of days with weather phenomena for the Tmax and the Tmin in each city, the number of days with a Tmin of 25 °C or higher (the criterion of tropical nights in Korea) increased significantly in Incheon. In Table 4, the p90days and the p99days of Incheon decreased or did not change, while in other cities they increased. In Incheon, Tmin25days increased by 297%. Incheon is located on the coast, the average Rh is higher than that of other cities and the Rh has increased compared to the past (Table 1). Rh and Tmin affect health as much as Tmax, they are meteorological factors used in predicting the impact of heatwaves in the United Kingdom, France, and the United States^42–44^. Therefore, an increase in relative risk in Incheon could be related to an increase in the Tmin and Rh.

**Table 4:**
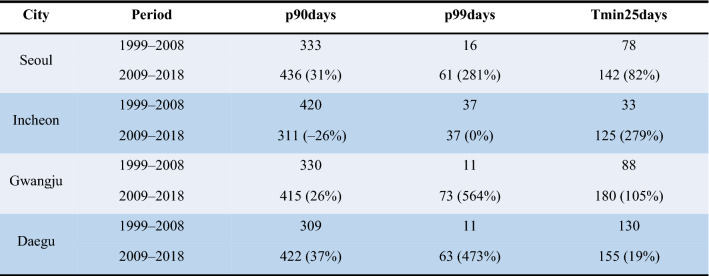
The number of days that exceed Tmax and Tmin thresholds, where ( ) indicates % change between decades.

p90dyas: number of days when the Tmax is above 90 percentile of Tmax (1999–2018).

p99dyas: number of days when the Tmax is above 99 percentile of Tmax (1999–2018).

Tmin25days: number of days when the temperature does not fall under 25 °C during the day.

Daegu has the highest proportion of the elderly among the study regions, and the proportion of low-income groups is 1.5 times that of Incheon. The average Tmax is also the highest (Table 1). Table 5 shows the number of doctors per 1,000 population and life expectancy in each city. The ratio of doctors and life expectancy are the highest in Seoul. Daegu's ratio of doctors is 2.2, the second lowest after Incheon, and its life expectancy is the lowest among all regions. However, in the vulnerable groups, the RR decreased significantly compared to the past. The interest in heatwave response was high in Daegu, the city where the heatwave warning service system was first installed in Korea. Seminars and conferences related to heatwave are held annually by the government and Non-governmental organizations. The results of Daegu's analysis show that policy responses can reduce the impact of heatwaves despite the magnitude of hazards and exposure.

**Table 5:** Average characteristics for each region in 2017–2018.

	Seoul	Incheon	Gwangju	Daegu
Number of doctor per population (1,000 people)	3.6	2.0	3.1	2.2
Life expectancy	83.4	81.7	81.8	81.6

Number of doctor per population^50^; life expectancy^46^.

## Conclusion

Many studies have shown that relative risk of heatwaves has generally decreased over time with adaptation, response policies, and socio-economic developments^17–20^. We also have found that the risks recently decreased in all age groups. According to Korea Power Exchange, the air conditioner supply rate in South Korea increased from 0.29 per household in 2000 to 0.60 in 2009 to 0.97 in 2019^45^. The life expectancy increased from 76.0 years in 2000 to 82.7 years in 2018^46^, implying that the individual health status and healthcare conditions have continued to improve^47^. Heatwave policies which have been implemented since 2008 may have contributed to reducing risks.

However, we have found that the risk of death from heat waves has increased in the socio-economically vulnerable group. The trend of increase was also seen in the elderly aged 65 and over. Increases of risks were more evident in single-person households. The single-person households in Korea have continuously increased from 23.6% of the total population in 2010 to 30.4% in 2020^48^. The increase in single-person households is not limited to Korea. According to Ortiz-Ospina, single-person households have approximately doubled in the past 50 years, and single-person households have continued to increase in many countries around the world^49^. This suggests that the socially isolated heat wave vulnerable population would increase in the future.

The high temperature-related risks in vulnerable groups tended to increase rapidly on Severe heat days. If the intensity and frequency of heat waves increase due to future climate change, the damage might be more concentrated in socio-economically vulnerable people. The risks of vulnerable groups have not increased in all cities with a high percentage of vulnerable groups or frequent heatwaves. Although it has unfavorable conditions in terms of heatwaves and the ratio of vulnerable groups, the risk has greatly decreased in Daegu, which has long been interested in heatwaves and aggressively implemented countermeasure policies. This suggests that interest and response policies for heatwaves play an important role in reducing damage.

This study suggests that efforts to resolve social polarization and social isolation will be a challenge to reduce future heatwave damage. The time-dependent increasing RR variation for the less-educated and single-person household groups indicated a benefit would arise from more customized heatwave warnings and impacts forecasting systems.

## Supplementary Information

Supplementary Information.

## Data Availability

The data that support the findings of this study are available from the MicroData Integrated Service (https://mdis.kostat.go.kr, South Korea) but restrictions apply to the availability of these data, which were used under license for the current study, and so are not publicly available. However, Koreans can download data by subscribing to the MicroData Integrated Service. A foreigner who is included as a joint researcher with Korean researchers when applying for data use can download the data. In addition, researchers and students belonging to an institution among foreigners residing in Korea with an alien registration card can download this data.
