# Supplementary material for: Analysis of time-dependent effects of ambient temperatures on health by vulnerable groups in Korea in 1999–2018
Source: Sci Rep. 2023 Jan 17;13:922. doi: 10.1038/s41598-023-28018-z (PMC9845373; doi:10.1038/s41598-023-28018-z)
Supplement: Supplementary file 1 — Supplementary Information. [file 41598_2023_28018_MOESM1_ESM.docx]

**Table S1. The annual mean number of deaths during summer (JJA) from 1999 to 2018 in each study city.**

| **City** | **All** | **Elderly** | **Single-person household** | **Less-educated** |
| --- | --- | --- | --- | --- |
| Seoul | 8443 | 5877 | 4117 | 5215 |
| Incheon | 2346 | 1600 | 1198 | 1629 |
| Gwangju | 1317 | 952 | 672 | 936 |
| Daegu | 2497 | 1752 | 1233 | 1826 |
| **Total**  (Percentage, %) | **14603**  (100.0) | **10180**  (69.7) | **7220**  (49.4) | **9607**  (65.8) |

**Table S2. Parameter values to generate a cross-basis matrix in each study city.**

| **City** | **Cross-basis function** | | | | | | | | |
| --- | --- | --- | --- | --- | --- | --- | --- | --- | --- |
|  | **All** | | **Elderly** | | **Single-person household** | | **Less-educated** | | |
|  | **Lag** | **Fun** | **Lag** | **Fun** | **Lag** | **Fun** | **Lag** | **Fun** |  |
| Seoul | 6 | ns | 6 | ns | 6 | poly | 6 | poly |  |
| Incheon | 6 | df=4 | 6 | poly | 6 | poly | 6 | poly |  |
| Gwangju | 4 | df=4 | 4 | df=4 | 4 | df=4 | 4 | poly |  |
| Daegu | 4 | poly | 3 | ns | 4 | df=4 | 4 | poly |  |

* Fun is a variable in the argvar list which is applied to vector of exposure (the daily maximum temperature in the study), in order to generate the matrix for the space of the predictor.

* The df is the degrees of freedom of a natural cubic spline in a constraining lag structure.

**
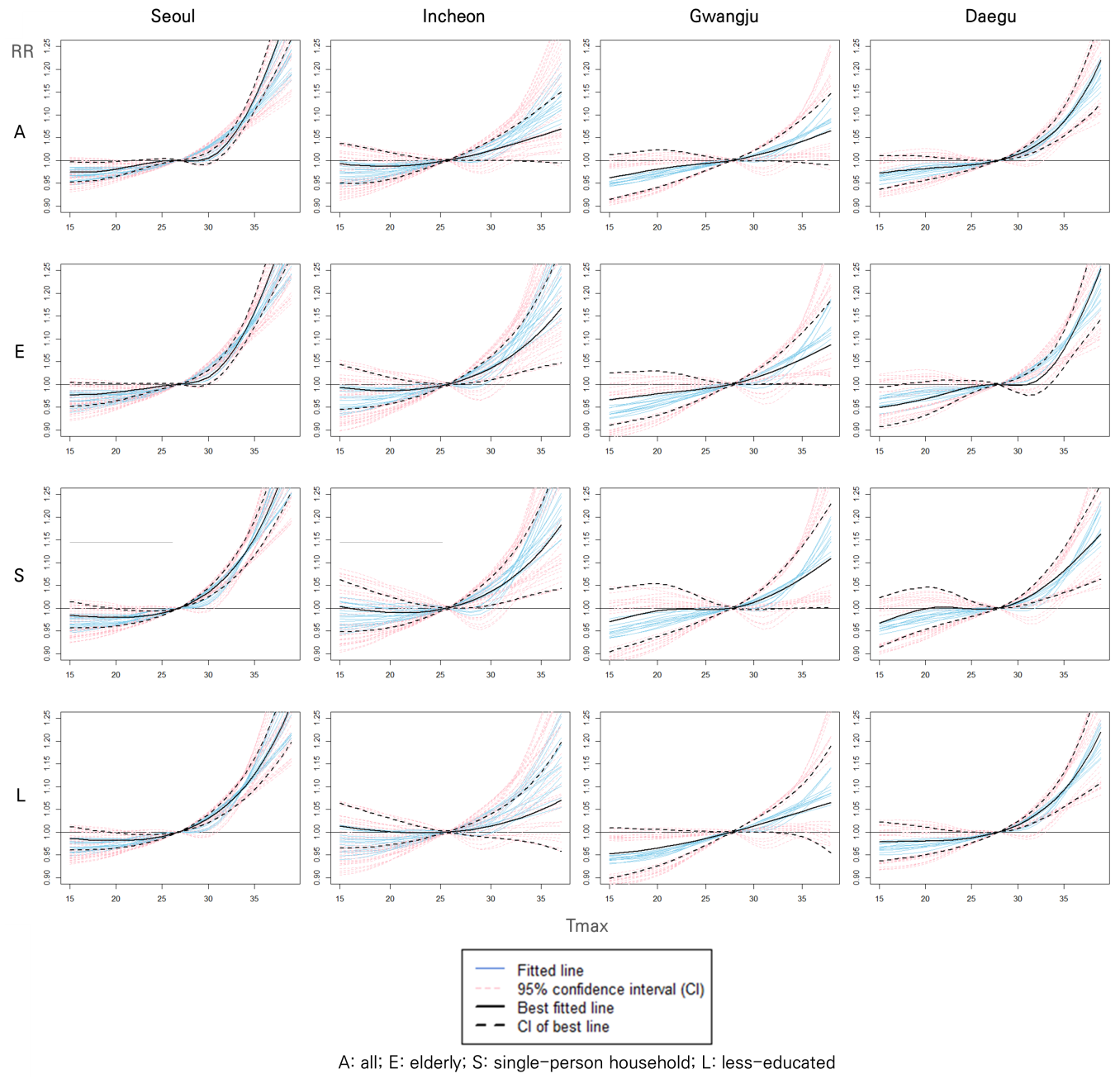
**

**Figure S1. Comparison of association between mortality and temperature at different lags and funs in each vulnerable group, 1999-2018. Thirty-six models constructed according to lags and funs were compared. The model with the smallest AIC was selected as the best fitted line.**
